# Technological and methodical process integration in manufacturing of monoclonal antibodies

**DOI:** 10.1186/1753-6561-9-S9-P39

**Published:** 2015-12-14

**Authors:** Petra Gronemeyer, Jochen Strube

**Affiliations:** 1 Institute for Separation and Process Technology, Clausthal University of Technology, 38678 Clausthal-Zellerfeld, Germany

## Background

A steady increase of product titers and the corresponding change in impurity composition represent a challenge for development and optimization of antibody production processes[1,2]. Concentration and composition of impurities like host cell proteins (HCP) are critical for efficient process development. These impurities show significant variations, which primarily depend on cell culture conditions. They have a strong influence on the downstream processing (DSP) and costs [2]. It is already shown that not optimized changes of parameters in USP can change the impurity profile by a factor of 7 [3]. The resulting "bottleneck" in DSP requires new optimization, technology and development approaches. These include the optimization and adaptation of existing unit operations respective to the new separation task, the assessment of alternative separation technologies and the search for new methods in process development [2].

## Material and Methods

By changing cell culture media compositions according to DoE screening designs, influences of media components on the HCP concentration and composition are identified. A media optimization is carried out regarding not only high product titers and cell growth but also HCP concentration and composition. By these experiments, different fermentation broths are produced containing different concentrations and compositions of HCP. These broths are used in subsequent development of ATPE and precipitation. A DoE based screening is carried out to identify the influence of following parameters: system composition, NaCl, cell number, viability, pH, product concentration, precipitant concentration, temperature, precipitation environment.

## Results

ATPE can be used as cell harvest method. It separates more than 95 % of cells from the broth. Almost 100 % of the antibody can be transferred in the top phase. The product is concentrated within the target phase at reduced volumes. At the same time, a first purification step is carried out removing HCP, DNA and media components. A subsequent precipitation removes the majority of remaining HCP and other impurities. The integration of ATP and precipitation can result into a significant reduction of costs and efforts. An optimization of the overall manufacturing process can be advanced by regarding process depending impurities resulting from the fermentation process. The optimization of USP towards relatively high product titers as well as an impurity spectrum which is easy to separate from the product can improve the manufacturing process concerning the total process costs.

## Conclusions

A combination of an integration of upstream and downstream processing regarding process depending impurities as well as an establishment and integration of alternative separation mechanisms can present an interesting solution to purify high concentrated antibodies.
